# Can Aquatic Plants Keep Pace with Climate Change?

**DOI:** 10.3389/fpls.2017.01906

**Published:** 2017-11-03

**Authors:** Duarte S. Viana

**Affiliations:** German Centre for Integrative Biodiversity Research (iDiv) Halle-Jena-Leipzig, Leipzig, Germany

**Keywords:** species distribution model, range shift, long distance dispersal, climate change, aquatic ecosystems, seed dispersal, migratory birds

## Abstract

The persistence of species may depend upon their capacity to keep pace with climate change. However, dispersal has been ignored in the vast majority of studies that aimed at estimating and predicting range shifts as a response to climate change. Long distance dispersal (LDD) in particular might promote rapid range shifts and allow species to track suitable habitat. Many aquatic plant species are dispersed by birds and have the potential to be dispersed over hundreds of kilometers during the bird migration seasons. I argue that such dispersal potential might be critical to allow species to track climate change happening at unprecedented high rates. As a case study, I used dispersal data from three aquatic plant species dispersed by migratory birds to model range shifts in response to climate change projections. By comparing four dispersal scenarios – (1) no dispersal, (2) unlimited dispersal, (3) LDD < 100 km, and (4) LDD mediated by bird migratory movements –, it was shown that, for bird-mediated dispersal, the rate of colonization is sufficient to counterbalance the rate of habitat loss. The estimated rates of colonization (3.2–31.5 km⋅year^-1^) are higher than, for example, the rate of global warming (previously estimated at 0.42 km⋅year^-1^). Although further studies are needed, the results suggest that these aquatic plant species can adjust their ranges under a severe climate change scenario. Therefore, investigating the dispersal capacity of species, namely their LDD potential, may contribute to estimate the likelihood of species to keep pace with climate change.

## Introduction

The rate at which climate is changing might preclude many species from adapting to novel conditions or moving to other areas where environmental conditions become suitable (Parmesan, 2006). Thus, whether species will be able to keep pace with climate change and ultimately persist is an urgent and crucial question in ecology, evolution and conservation science. Evidence already suggests that many species can adjust their geographic ranges in response to climate change (Parmesan, 2006; Chen et al., 2011; Lenoir and Svenning, 2015). However, the lack of knowledge and integration of dispersal for most species is hampering a general assessment of the potential of species to track climate change (Bateman et al., 2013).

Many species might have dispersal capacities that allow them to adjust their ranges under current and future rates of climate change, namely those that have a means to engage in long distance dispersal (LDD) (e.g., Engler and Guisan, 2009; Gillespie et al., 2012; Corlett and Westcott, 2013; Viana et al., 2016). This has been increasingly recognized, and recent platforms for modeling species distributions have included dispersal as a core process in species range shifts (Zurell et al., 2016). LDD is defined here as dispersal beyond both the genetic neighborhood and population boundaries (i.e., strict-sense LDD; Jordano, 2017). However, only a very low proportion of studies on changes in species distributions, and thus of modeled species, incorporated dispersal data into their models (Bateman et al., 2013). This is precluding our ability to more realistically understand whether species will be able to keep pace with climate change and occupy suitable habitat in the future (Thuiller et al., 2008; Bateman et al., 2013; Hellmann et al., 2016). Which species have this capacity is thus an important question, though it remains largely unexplored.

In the aquatic realm, dispersal is even a more determinant factor due to the patchy nature of inland aquatic ecosystems (Heino, 2011; Bush and Hoskins, 2017). Aquatic plants, but also other aquatic organisms such as algae and invertebrates, are able to disperse overland through dispersal of seeds and other propagules by birds (Figuerola and Green, 2002; Brochet et al., 2009; van Leeuwen et al., 2012). Many waterbirds are migratory, and can regularly disperse propagules over distances that can potentially allow these aquatic organisms to achieve high movement and colonization rates (Viana et al., 2016). Evidence for seed dispersal by migratory birds comes from waterbird diet studies, which showed that a great diversity of waterbird species consume and deposit large amounts of a variety of seed species (e.g., van Leeuwen et al., 2012); and from mechanistic dispersal models that combine waterbird movements and seed retention times in or on the birds’ body (e.g., Viana et al., 2013; Kleyheeg et al., 2017). This dispersal capacity concur with the generally broad distributional ranges of aquatic plants (Santamaría, 2002), and with gene flow occurring over large scales (King et al., 2002). Although many factors might reduce seed dispersal effectiveness (SDE), such as phenological mismatches between migration and seed production, unidirectionality during bird migration, and the unsuitability of arrival habitats (Clausen et al., 2002), all the latter studies support that waterbirds are effective dispersers of aquatic plant seeds. There is accumulating evidence for both the quantity and quality of seed dispersal that suggests high SDE, namely the large amounts of seeds dispersed (an average of 45% of sampled waterbird droppings contain seeds) that remain viable following transportation (36% on average; van Leeuwen et al., 2012). For a more profound review of SDE, see Viana et al. (2016).

Despite the LDD potential of aquatic plants and their important role for the maintenance of aquatic biodiversity and provision of ecosystem services, present and future distribution patterns remain poorly studied in comparison with terrestrial plants (Kosten et al., 2009; Alahuhta et al., 2011; Feeley et al., 2017). Some examples of projections into the future include the invasive potential of some aquatic plants and the forecast of probable invasion areas (Gillard et al., 2017), as well as projections in particular biomes or regions, such as in boreal systems (Heino et al., 2009; Alahuhta et al., 2011) and in Oceania (Bush and Hoskins, 2017). In the latter study, the importance of dispersal is also highlighted, although dispersal by migratory birds was not included in their estimations.

Species distribution models (SDMs) are widely used as forecasting tools (e.g., Guisan et al., 2013). These models allow to relating past and present climatic conditions to the presence-absence of species, and predict their potential presence for a projected climate scenario. However, these models often assume a scenario of either none or unlimited dispersal, and thus the probable future distribution will lie somewhere between these two extreme scenarios (Bateman et al., 2013). To exemplify and investigate whether aquatic plants will keep pace with climate change, I followed the basic framework presented in Viana et al. (2016), implemented using the MigClim model (Engler et al., 2012), to compare different dispersal scenarios in three aquatic plant species: (1) no dispersal, (2) seed dispersal by local bird movements (<100 km; hereafter “local dispersal”), (3) seed dispersal by local and migratory bird movements (hereafter “full LDD”), and (4) unlimited dispersal. The latter corresponds to a situation in which the species would fully track available habitat.

## Case Study: Range Shifts of Three Aquatic Plant Species

I modeled range dynamics for three aquatic plant species: fennel pondweed *Stuckenia pectinata* (L.) Börner, common club-rush *Schoenoplectus lacustris* (L.) Palla and sea club-rush *Bolboschoenus maritimus* (L.) Palla. Their initial (present) distributions were obtained from presence records downloaded from the Global Biodiversity Information Facility (GBIF) and cropped to their European native range, where dispersal data was available (see section Dispersal models). Although GBIF records are probably incomplete and may contain inaccuracies, it should have a minor impact on the broad-scale dynamics modeled here. These species were chosen because there are available data for seed gut retention time and posterior germinability. I used the MigClim modeling platform in R (Engler et al., 2012) to simulate range shifts according to the dispersal scenarios presented above. As a first step, I estimated a SDM using bioclimatic variables averaged over the past 40 years. Then, using climate projections and published dispersal data, I simulated range shifts for the next 80 years by updating climatic data every 10 years. I performed 10 simulation replicates to control for stochasticity in the dispersal process. Due to their plasticity and adaptability (Santamaría et al., 2003), I assumed that these species can produce propagules during the first season after colonization, and that in the second year the plants reach full reproductive potential. Detailed methods can be seen in the sections below and the full R code is available in Supplementary Material. The simulations can be fully reproduced using the R code.

### Species Distribution Model

The distribution and community structure of aquatic plant species are strongly determined by climatic variables, such as temperature and precipitation, and their top-down effects on habitats at the landscape and local scales (Dudgeon et al., 2006; Heino et al., 2009; Kosten et al., 2009; Alahuhta et al., 2011). Although climate projections are calibrated mainly for atmospheric conditions, a high proportion of water availability depends on precipitation regimes. Moreover, air temperature was found to be correlated with water temperature across the globe (Sharma et al., 2015). Therefore, I used commonly used bioclimatic variables to model species distributions. Note, however, that the objective was to assess the role of bird-mediated dispersal on the rate of expansion, and not to accurately predict species distributions.

The SDM was estimated using Maxent from the R package “dismo” (Hijmans et al., 2017) with bioclimatic variables obtained from the WorldClim database (Hijmans et al., 2005). This model was used to predict habitat suitability under a climate change scenario with severe greenhouse gas emissions. Future climate data downscaled to 10 arc minutes (according to the Delta Method of IPCC AR4) was downloaded from the CCAFS-Climate data portal using the R package “ccafs” (Chamberlain, 2017). The decanal availability of downscaled data constrained the choice of climate change scenario – I used the Global Circulation Model “mpi_echam5” (developed at the Max Planck Institute for Meteorology) with the emissions scenario “A2” (which predicts temperature increases of 2.0–5.4°C by 2100) for the period 2020–2090. Besides bioclimatic variables, only cells where water was present were defined as suitable. The presence of water was obtained from the Global Lakes and Wetlands Database (WWF, the Center for Environmental Systems Research, University of Kassel, Germany). To avoid collinearity and model overfit in the SDM, bioclimatic variables were selected by using a backward selection procedure in which variables that were less important to explain species presence and had correlation coefficients >0.70 were first excluded until all pairwise correlations were below 0.70. Model performance was assessed using cross-validation and the ROC curve area between training and test data. The most important variable for all species was the temperature annual range, except for *B. maritimus*, which was temperature seasonality. Other important variables common to all species included precipitation seasonality and mean temperature of wettest quarter. Common variables to at least two species included the mean temperature of driest quarter and minimum temperature of coldest month (**Table 1**). Model performance ranged from 0.91 to 0.96, as estimated by the ROC curve area corresponding to the Somers’ Dxy rank correlation between training and test data.

**Table 1:** Summary results from the SDM (selected bioclimatic variables), dispersal kernels and range shift models (MigClim).

	*Stuckenia pectinata*	*Schoenoplectus lacustris*	*Bolboschoenus maritimus*
Selected niche variables	T annual range, P driest month, min T coldest month, min T driest quarter, P seasonality, isothermality, mean T wettest quarter	T annual range, P driest quarter, max T warmest month, mean T driest quarter, mean T wettest quarter, P seasonality	T seasonality, min T coldest month, annual P, P seasonality, mean T wettest quarter
Dispersal vectors	Mallard	Mallard	Mallard, Teal
Max dispersal distance (km)	593	1285	2648
Frequency (%) of LDD > 100 km	0.06	0.15	0.41
Extinction rate (km⋅year^-1^)			
Local dispersal	1.9	3.2	0.9
Local+LDD	4.1	7.3	7
Colonization rate (km⋅year^-1^)			
Local dispersal	3.2	8	3.2
Full LDD	12.1	19.8	31.5
Expansion rate (km⋅year^-1^)			
Local dispersal	1.3	3.5	1.5
Full LDD	5.6	12.5	28.5

*P, precipitation; T, temperature*.

### Dispersal Models

Seed dispersal for the three aquatic plant species was mediated by mallards (*Anas platyrhynchos*) and teals (*A. crecca*), two abundant waterfowl species that migrate each year across Europe. Dispersal kernels were estimated by combining movement distances with gut retention times according to a procedure described in Viana et al. (2013). I estimated for each aquatic plant species a local dispersal kernel (<100 km) and a full LDD kernel using the appropriate movement distance parameters obtained from the same publication. These parameters were based on empirical data, providing a more realistic set for the range dynamics simulation. It is worth noting that allometric scaling can be used to derive dispersal kernels for other migratory bird vectors and other aquatic seeds to further explore the responses of aquatic vegetation to climate change (Viana et al., 2013, 2016). The difference between local and migratory dispersal kernels lay in the maximum distances (100 km for local distances, and from 500 km to >1000 km for migratory distances, depending on the species; **Table 1**). The frequency of LDD > 100 km ranged from 0.06 to 0.41% (**Table 1**).

### Predicted Future Distributions under the Different Dispersal Scenarios

**FIGURE 1:**
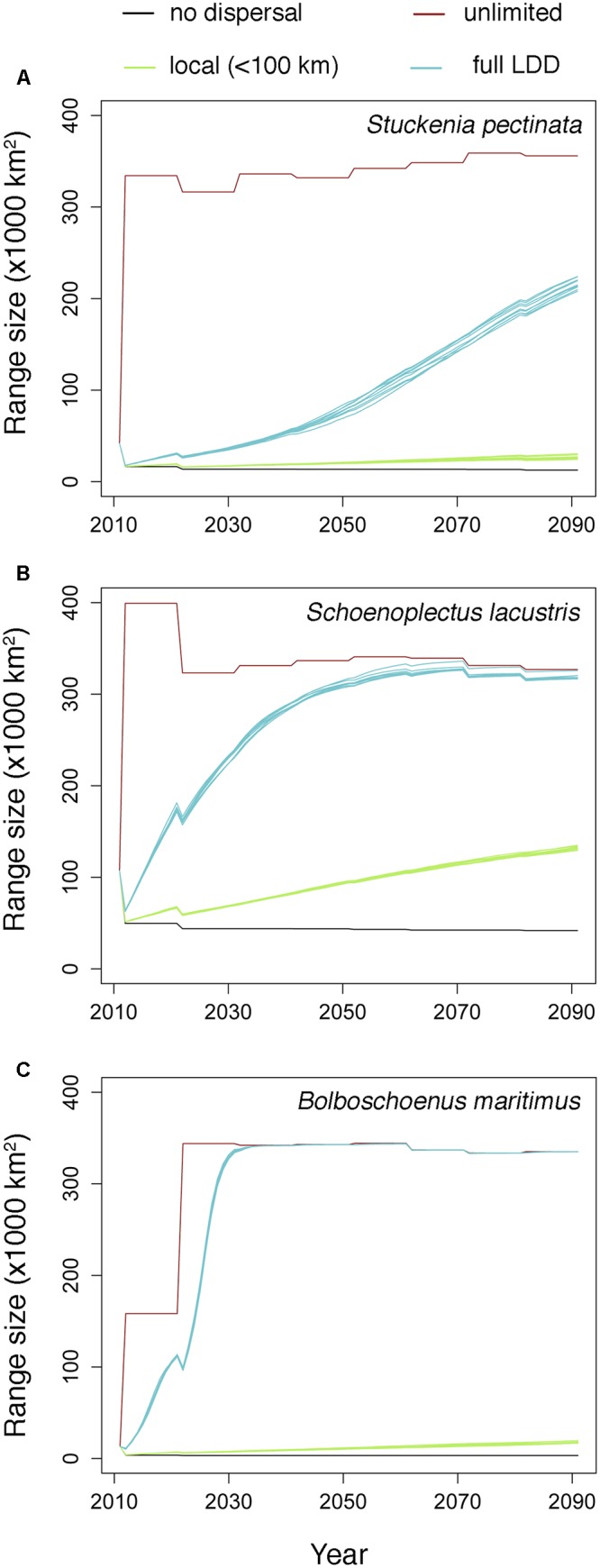
Estimated range expansion for the three aquatic plant species under climate change according to the different dispersal scenarios. The multiple lines for each dispersal scenario (where appreciable) correspond to simulation replicates (*N* = 10). **(A)** *Stuckenia pectinata*; **(B)** *Schoenoplectus lacustris*; **(C)** *Bolboschoenus maritimus*.

Habitat suitability in original occurrence areas was reduced by climate change, resulting in the contraction of the distributions of all species when they could not disperse. However, when species were allowed to disperse, their distribution ranges expanded to areas where climate became suitable (**Figure 1**). The rate of expansion differed according to the dispersal scenario (**Figure 1** and **Table 1**). When dispersal vectors were allowed to move only over local distances (<100 km), colonization rates ranged from 3.3 to 8.0 km⋅year^-1^, but when migratory movements were included in the dispersal kernels (full LDD), expansion rates ranged from 12.1 to 31.5 km⋅year^-1^ (as calculated by the squared root of the average annual colonized area). In both cases, the colonization rates were higher than the local extinction rates, resulting in net range expansion for all species (**Figure 1** and **Table 1**). For all but one species, the distribution ranges eventually reached virtually all the available area after 20–40 years, showing the power of full LDD in promoting range expansion. For the pondweed *Stuckenia pectinata*, however, the colonization rate was lower, and so was the rate of convergence between habitat availability and species distribution, as this species shows relatively shorter seed retention times in the birds’ body and thus lower frequency of LDD > 100 km (**Table 1**).

The estimated rates of colonization should be sufficient to allow species to track climate suitability. For example, global temperature is predicted to be increasing at a mean velocity of 0.42 km⋅year^-1^ (0.11–0.46 km⋅year^-1^) (Loarie et al., 2009); hence, aquatic plants dispersed by birds may be able to keep pace with climate change. Altogether these results suggest that quantifying the dispersal capacity may be critical for predicting the likelihood of species to adjust their ranges under the relatively high rate of climate change. Studying the mechanisms underlying long distance seed dispersal is one possible approach (Nathan et al., 2008).

For the studied aquatic plants, the traits that allow for such dispersal capacities are related to seed characteristics, as well as the phenology of seed production and formation of propagule banks, that might maximize the probability of encounter with dispersal vectors (Viana et al., 2016). In other taxa, e.g., whose seeds are dispersed by water or wind, or birds with a lower movement capacity, the ability to keep pace with climate change might be reduced (Nathan et al., 2008). However, many studies have concluded that seed dispersal estimates are not sufficient to explain the faster-than-expected plant migration rates estimated from historical records – as illustrated by the Reid’s paradox (Clark et al., 1998). Indeed, seed dispersal in general has been estimated to occur within 10–1500 m (Corlett and Westcott, 2013), which might be insufficient to explain both post-glacial migration rates (e.g., estimates for trees range from 0.06 to 0.5 km⋅year^-1^; Feurdean et al., 2013) and current latitudinal shifts of terrestrial species, including plants, estimated to be occurring at a median rate of 1.7 km⋅year^-1^ (Chen et al., 2011). Therefore, evidence suggests that organisms in general have higher dispersal capacities than usually estimated. This might be due to the fact that LDD events (e.g., beyond 1.5 km) are difficult to quantify and often underestimated in dispersal models.

The results from this simulation model show the importance of studying and estimating dispersal potential to predict which species will be able to migrate without further assistance. In aquatic ecosystems, aquatic plants that produce seeds that can survive transportation by waterbirds are good candidates to keep pace with climate change, especially if their seeds form abundant banks in the sediment where many waterbirds species feed on. This maximizes the possibility of both ingestion and consequent endozoochory, and attachment to the birds’ body and consequent ectozoochory. For other plants and organisms, traits such as fruiting during bird migration seasons, evolvability of dispersal capacity at the range front, body size in animals, among others can favor the likelihood of LDD (Nathan et al., 2008; Viana et al., 2016).

## Caveats and Future Developments

There is an extensive literature on SDMs, and one of their main limitations is the characterization of the species’ niche, in particular when predicting range shifts based on climate change. The model presented here only accounted for climatic variables and presence of water, though it is known that many other local habitat variables are important to explain species presence, such as water chemistry. Some parameters related to water chemistry, such as nutrient concentrations (e.g., phosphorus), might even be influenced by climatic variables (e.g., Jeppesen et al., 2009), and thus these effects should be taken into account when predicting habitat suitability for the future. Nevertheless, aquatic plants have in general quite broad distributions, in many cases cosmopolitan distributions (Santamaría, 2002), which point to the role of plasticity and local adaptation. Indeed, Santamaría et al. (2003) showed that the pondweed *Stuckenia pectinata* (one of the species used here) can survive and reproduce across distant latitudes in Europe varying in climate but also in other habitat characteristics. Another line of evidence comes from a recent study showing that the niche of aquatic plants is not conserved among different regions (Alahuhta et al., 2017). Although this might imply that assumptions regarding habitat suitability can be relaxed, it also affects the estimation of SDMs, whose predictions can be less generalized across increasing spatial scales. Thus regional idiosyncrasy in species niches should be probably taken into account when modeling species distributions.

Another important, but often neglected, factor when predicting range shifts is the directionality of dispersal. Because climate change is in itself directional, since change is more pronounced across latitude than across longitude, if the direction of dispersal is not coincident with that of climate change, the ability to expand or shift might be decoupled. Birds mostly migrate along a latitudinal gradient twice a year, by moving to northern latitudes during the spring migration and to southern latitudes during the autumn migration. However, these movements occur along preferential migration routes that might not, or only partially, overlap with suitable habitat for plants, and thus it is important to incorporate movement direction into range shift models. Adding this feature to modeling platforms such as MigClim (see also Zurell et al., 2016) would thus be a useful tool for many species with directional LDD, including species dispersed by wind, water and animals, as well as vagile organisms such as migratory animals (Nathan et al., 2008; Gillespie et al., 2012). Moreover, climate change might affect bird future distributions, migratory movements and physiology, and lead to changes in seed dispersal. It will be important to assess to what extent the effects of climate change on seed dispersal will affect the ability of aquatic plants to migrate and adjust their ranges.

## Conclusion

Can aquatic plants keep pace with climate change? Although this article cannot directly answer this question, I provide arguments, evidence and tools suggesting that many aquatic plants dispersed by migratory birds can potentially expand fast enough to be able to reach suitable habitat in the future. The results from the range shift models show the importance of integrating dispersal capacity with habitat suitability to predict future species distributions. Because LDD accelerates the rate of range shifts, refining estimates of LDD by studying relevant species traits and underlying mechanisms will aid in identifying the species that might keep pace with climate change versus those that might need assistance, for example through conservation plans. Dispersal by migratory birds can be a predictable phenomenon, and thus more reliable predictions of range shifts might be achieved for the many species that may use their services to disperse.

## Author Contributions

DV conceived the idea, performed all the analyses, and wrote the manuscript.

## Conflict of Interest Statement

The author declares that the research was conducted in the absence of any commercial or financial relationships that could be construed as a potential conflict of interest.
