# Supplementary material for: Can Aquatic Plants Keep Pace with Climate Change?
Source: Front Plant Sci. 2017 Nov 3;8:1906. doi: 10.3389/fpls.2017.01906 (PMC5701636; doi:10.3389/fpls.2017.01906)
Supplement: Supplementary file 1 [file Data_Sheet_1.PDF]

Supplementary Material

Viana 2017

```
##############################
## Duarte S. Viana, 2017 ###
#############################

# Code for simulating range shifts of species dispersed by migratory birds under climate change
# Example for one aquatic plant species: Scirpus maritimus
# Note that before the code is run, water data must be manually downloaded

############################################################################
############################################################################

# Load libraries
library(raster)
library(dismo)
library(rms)
library(rgdal)
library(plotrix)
library(mapdata)
library(plyr)
library(distr)
distroptions("TruncQuantile"=1e-8)

# Define study extent (geographical coordinates for the crop area)
clip<-matrix(c(-24, 45, 27, 72), nrow = 2, ncol = 2, byrow = TRUE,
        dimnames = list(c("x", "y"),c("min", "max")))


############################################################################
############################################################################

# 1. Data gathering


# 1.1. Download species presence data from GBIF
# Data should be chacked and cleaned
sm<-gbif('scirpus', 'maritimus', sp=TRUE, removeZeros=TRUE)
# plot records in map
map('worldHires')
points(sm, col='red')

# 1.2. Import raster with bioclimatic data from 1970-2000
# use the getData function from the raster package to download WorldClim climate data
# only for the WorldClim 1.4 version
climpres<-getData('worldclim', var='bio', res=10)
names(climpres)<-
c("bio01","bio02","bio03","bio04","bio05","bio06","bio07","bio08","bio09","bio010",
```

```
                "bio11","bio12","bio13","bio14","bio15","bio16","bio17","bio18","bio19")

# 1.3. Download future climate data (bioclimatic), downscaled for 10 min of degree
library(ccafs)

# 2020s
key<-cc_search(file_set = 4, extent = "global", format = "ascii", scenario = 5, model =
30, period = 3, variable = 1, resolution = 4)
res <- cc_data_fetch(key = key)
clim2020s<-cc_data_read(res)
names(clim2020s)<-
c("bio01","bio02","bio03","bio04","bio05","bio06","bio07","bio08","bio09","bio010",
                "bio11","bio12","bio13","bio14","bio15","bio16","bio17","bio18","bio19")
clim2020s<-crop(clim2020s,clip)
plot(clim2020s,"bio01") # up to 19 variables

# 2030s
key<-cc_search(file_set = 4, extent = "global", format = "ascii", scenario = 5, model =
30, period = 4, variable = 1, resolution = 4)
res <- cc_data_fetch(key = key)
clim2030s<-cc_data_read(res)
names(clim2030s)<-
c("bio01","bio02","bio03","bio04","bio05","bio06","bio07","bio08","bio09","bio010",
                "bio11","bio12","bio13","bio14","bio15","bio16","bio17","bio18","bio19")
clim2030s<-crop(clim2030s,clip)
plot(clim2030s,"bio01") # up to 19 variables

# 2040s
key<-cc_search(file_set = 4, extent = "global", format = "ascii", scenario = 5, model =
30, period = 5, variable = 1, resolution = 4)
res <- cc_data_fetch(key = key)
clim2040s<-cc_data_read(res)
names(clim2040s)<-
c("bio01","bio02","bio03","bio04","bio05","bio06","bio07","bio08","bio09","bio010",
                "bio11","bio12","bio13","bio14","bio15","bio16","bio17","bio18","bio19")
clim2040s<-crop(clim2040s,clip)
plot(clim2040s,"bio01") # up to 19 variables

# 2050s
key<-cc_search(file_set = 4, extent = "global", format = "ascii", scenario = 5, model =
30, period = 6, variable = 1, resolution = 4)
res <- cc_data_fetch(key = key)
clim2050s<-cc_data_read(res)
names(clim2050s)<-
c("bio01","bio02","bio03","bio04","bio05","bio06","bio07","bio08","bio09","bio010",
                "bio11","bio12","bio13","bio14","bio15","bio16","bio17","bio18","bio19")
clim2050s<-crop(clim2050s,clip)
plot(clim2050s,"bio01") # up to 19 variables
```

```
# 2060s
key<-cc_search(file_set = 4, extent = "global", format = "ascii", scenario = 5, model =
30, period = 7, variable = 1, resolution = 4)
res <- cc_data_fetch(key = key)
clim2060s<-cc_data_read(res)
names(clim2060s)<-
c("bio01","bio02","bio03","bio04","bio05","bio06","bio07","bio08","bio09","bio010",
            "bio11","bio12","bio13","bio14","bio15","bio16","bio17","bio18","bio19")
clim2060s<-crop(clim2060s,clip)
plot(clim2060s,"bio01") # up to 19 variables

# 2070s
key<-cc_search(file_set = 4, extent = "global", format = "ascii", scenario = 5, model =
30, period = 8, variable = 1, resolution = 4)
res <- cc_data_fetch(key = key)
clim2070s<-cc_data_read(res)
names(clim2070s)<-
c("bio01","bio02","bio03","bio04","bio05","bio06","bio07","bio08","bio09","bio010",
            "bio11","bio12","bio13","bio14","bio15","bio16","bio17","bio18","bio19")
clim2070s<-crop(clim2070s,clip)
plot(clim2070s,"bio01") # up to 19 variables

# 2080s
key<-cc_search(file_set = 4, extent = "global", format = "ascii", scenario = 5, model =
30,period = 9, variable = 1, resolution = 4)
res <- cc_data_fetch(key = key)
clim2080s<-cc_data_read(res)
names(clim2080s)<-
c("bio01","bio02","bio03","bio04","bio05","bio06","bio07","bio08","bio09","bio010",
            "bio11","bio12","bio13","bio14","bio15","bio16","bio17","bio18","bio19")
clim2080s<-crop(clim2080s,clip)
plot(clim2080s,"bio01") # up to 19 variables



################################################################################
################################################################################

# 2. Species distribution model (SDM)


# clip data to the study extent
# Presence data
sm2 <- crop(sm,clip)
sm2@data[,1]<-1
names(sm2@data)<-"presence"
# climatic data
climpres<-crop(climpres,clip)

# plot data
```

```
plot(climpres,"bio01")
points(sm2,cex=0.5)

# Selecting background points
set.seed(23)
background <- randomPoints(climpres, 5000)
background <- as.data.frame(background)
background$presence <- 0

# Combine presence and background
pres_points <- as(sm2, "data.frame")
colnames(pres_points) <- c("presence","x","y")
pres_bg <- rbind(pres_points[,colnames(background)], background)
coordinates(pres_bg) <- ~ x + y
proj4string(pres_bg) <- proj4string(sm2)

# Add environmental data
bioclim<-extract(climpres, pres_bg)
pres_bg@data <- cbind(pres_bg@data, bioclim)
pres_bg <- as(pres_bg, "data.frame")
pres_bg <- na.omit(pres_bg) # remove NAs

# Collinearity
bioclim<-pres_bg[, 4:ncol(pres_bg)]
round(cor(bioclim), 2)
# If variables are highly correlated (e.g. >0.70), perform variable selection (see
manuscript)
# variable selection for S. maritimus
pres_bg<-cbind(pres_bg[,c(1:3)],bioclim[,-c(1,13,5,14,16,19,17,2,3,7,9,10,18,11)])

# Perform the SDM with Maxent
# Maxent is a Java program that needs to be installed separately
# (check documentation of package "dismo")
me_presbg <- maxent(pres_bg[, 4:ncol(pres_bg)], pres_bg[, "presence"])

# Variable importance
plot(me_presbg)

# Model performance: crossvalidation
set.seed(2)
fold <- kfold(pres_bg, k = 5, by = pres_bg$presence)
# The variable cv_pred will contain the cross-validated data
# predictions
pres_bg$cv_pred <- NA
for (i in unique(fold)) {
  traindata <- pres_bg[fold != i, ]
  testdata <- pres_bg[fold == i, ]
  cv_model <- maxent(traindata[, 4:(ncol(traindata)-1)], traindata[, "presence"])
  pres_bg$cv_pred[fold == i] <- predict(cv_model, testdata, args='outputformat=raw')
}
```

```
# ROC area
round(somers2(pres_bg$cv_pred, pres_bg$presence), 2)
round(somers2(predict(me_presbg, pres_bg, args='outputformat=raw'),
pres_bg$presence), 2)
# Quite good performance between training and test data.

# Remove validation column
pres_bg<-pres_bg[,-which(names(pres_bg)=="cv_pred")]


##############################################################################
##############################################################################

# 3. Estimate dispersal kernels according to Viana et al. (2013; American Naturalist,
181: 649–62)
# Parameter values were taken from the same publication

# Scirpus maritimus
pars<-
data.frame(Fmig=NA,loc.mean=NA,loc.sd=NA,mig.mean=NA,mig.sd=NA,f.speed=N
A, grt.mean=NA,grt.sd=NA,F0=NA,dlt=NA)
# Parameters according to vector species
pars[1,]<-c(0.08, 2.42, 0.95, 5.30, 0.64, 73.8, 1.56, 0.56, 0.05, 1) # A. platyrhynchos
pars[2,]<-c(0.19, 2.51, 0.96, 5.72, 0.68, 74.16, 2.41, 0.58, 0.05, 1) # A. crecca

# Run model for local and migratory movements
disp.curve<-list()
for(i in 1:nrow(pars)) {
  set.seed(999)
  # number of dispersed seeds
  Q<-1000000
  # frequency of movement
  Fmov<-Q*(pars$F0[i])
  m0<-rep(0,Q-Fmov)
  # distance distribution
  mix.mov<-UnivarMixingDistribution(Lnorm(pars$loc.mean[i],pars$loc.sd[i]),
             Lnorm(pars$mig.mean[i],pars$mig.sd[i]),mixCoeff=c(1-
pars$Fmig[i],pars$Fmig[i]))
  mix.mov.trunc<-Truncate(mix.mov,lower=0.001,upper=3000)
  # sample vector distances
  mov.sample<-r(mix.mov.trunc)(Fmov)
  mov<-c(m0,mov.sample)
  # distribution of seed rention times
  fit.grt<-Lnorm(pars$grt.mean[i],pars$grt.sd[i])
  fit.grt.trunc<-Truncate(fit.grt,lower=0.5,upper=52)
  # time until departure
  if(pars$dlt[i]==0) dlt<-r(Truncate(Exp(1.2),lower=0.5,upper=6))(Q)
  if(pars$dlt[i]==1) dlt<-r(Unif(0.5,6))(Q)
  if(pars$dlt[i]==2) dlt<-6-r(Truncate(Exp(1.2),lower=0,upper=5.5))(Q)
```

```
  # sample retention times
  grt<-r(fit.grt.trunc)(Q)-dlt
  grt[grt<0]<-0
  # estimate maximum distances based on flight speed
  Pmdd<-grt*pars$f.speed[i]
  # Estimate dispersal distances
  d.rand<-sample(mov,Q,replace=T)
  s.rand<-sample(Pmdd,Q,replace=T)
  if(length(which(s.rand==0))==0) s.0<-Q+1
  if(length(which(s.rand==0))>0) s.0<-which(s.rand==0)
  dist0<-s.rand[s.0]
  dist1<-ifelse(d.rand[-s.0]<=s.rand[-s.0],d.rand[-s.0],NA)
  if(length(which(s.rand==0))==0) dist<-dist1
  if(length(which(s.rand==0))>0) dist<-c(dist0,dist1)
  d.in<-na.exclude(dist)
  disp.dist<-d.in[d.in>0]
  disp.curve[[i]]<-d.in
}

# Combine dispersal kernels (from the two vectors)
total.disp<-unlist(disp.curve)

# Cumulative function
disp.cum<-ecdf(total.disp)

# estimate probability for each cell away from the dipersal origin
# cell size is 10 min ~ 1.852 km (1 nautical mile) *10
unit.class<-10*1.852
dclass<-seq(unit.class,max(total.disp),by=unit.class)
disp.prob<-1-disp.cum(dclass)


##########################################################################
##########################################################################

# 4. Simulate range expansion/shift with MigClim

library(MigClim)

# Species initial distribution [iniDist]
# Cells with species presence
pres.layer<-climpres[[1]]
pres.cells<-cellFromXY(pres.layer,sm2)
values(pres.layer)[pres.cells]<-1
values(pres.layer)[-pres.cells]<-0
plot(pres.layer)

# Habitat suitability map(s) [hsMap]
# Load future climate
```

```
climfut<-
list(clim2020s,clim2030s,clim2040s,clim2050s,clim2060s,clim2070s,clim2080s)

# Load water data
# Downloaded from https://www.worldwildlife.org/publications/global-lakes-and-
wetlands-database-lakes-and-wetlands-grid-level-3
setwd("~/GLWD-level3") # set working directory
water.layer<-raster("glwd_3")
water.layer<-crop(water.layer,clip)
# match resolution
# fact = res(climpres)/res(water.layer)
water.layer<-aggregate(water.layer,fact=20)
plot(water.layer)

# Estimate present suitability
pred0<-predict(me_presbg,climpres[[names(pres_bg)[-c(1:3)]]])
# rescale prediction raster values to 0-1000 (to use it in MigClim)
values(pred0)<-rescale(values(pred0),c(0,1000))
# habitat suitability for dry places is 0
values(pred0)[is.na(values(water.layer))]<-0
# Set NA to 0
values(pred0)[is.na(values(pred0))]<-0
# rescale again
values(pred0)<-round(rescale(values(pred0),c(0,1000)))

# Estimate habitat suitability for the future
# restricting suitability to water
hs.layers<-list(pred0)
for(i in 1:7){
  climfut.i<-climfut[[i]][[names(pres_bg)[-c(1:3)]]]
  pred<-predict(me_presbg,climfut.i)
  # rescale prediction raster values to 0-1000 (to use it in MigClim)
  values(pred)<-rescale(values(pred),c(0,1000))
  # habitat suitability for dry places is 0
  values(pred)[is.na(values(water.layer))]<-0
  # Set NA to 0
  values(pred)[is.na(values(pred))]<-0
  # rescale again
  values(pred)<-round(rescale(values(pred),c(0,1000)))
  hs.layers[[1+i]]<-pred
}


# Organize data in a data frame
data<-as.data.frame(coordinates(pres.layer))
data$IniDist<-values(pres.layer)
for(i in 1:length(hs.layers)) data[,3+i]<-values(hs.layers[[i]])
names(data)<-
c("Xcoordinate","Ycoordinate","IniDist","HSmap1","HSmap2","HSmap3","HSmap4","
HSmap5","HSmap6","HSmap7","HSmap8")
```

```
# Run model
mig.mod<-MigClim.migrate (iniDist=data[,1:3],
            hsMap=data[,4:ncol(data)], rcThreshold=10,
            envChgSteps=8, dispSteps=10, dispKernel=disp.prob,
            iniMatAge=1, propaguleProd=c(0.5),
            simulName="MigClim_Sm", replicateNb=10, overWrite=TRUE,
            testMode=FALSE, fullOutput=FALSE, keepTempFiles=FALSE)


############################## End ###################################
```
